# Wearable accelerometry-based technology capable of assessing functional activities in neurological populations in community settings: a systematic review

**DOI:** 10.1186/1743-0003-11-36

**Published:** 2014-03-13

**Authors:** Dax Steins, Helen Dawes, Patrick Esser, Johnny Collett

**Affiliations:** 1 Movement Science Group, Oxford Brookes University, Oxford, UK; 2 Department of Clinical Neurology, University of Oxford, Oxford, UK; 3 Cardiff University, Wales, UK

**Keywords:** Wearable technology, Telerehabilitation, Mobility, Accelerometers, Motion analysis, Home monitoring, Machine learning

## Abstract

**Background:**

Integrating rehabilitation services through wearable systems has the potential to accurately assess the type, intensity, duration, and quality of movement necessary for procuring key outcome measures.

**Objectives:**

This review aims to explore wearable accelerometry-based technology (ABT) capable of assessing mobility-related functional activities intended for rehabilitation purposes in community settings for neurological populations. In this review, we focus on the accuracy of ABT-based methods, types of outcome measures, and the implementation of ABT in non-clinical settings for rehabilitation purposes.

**Data sources:**

Cochrane, PubMed, Web of Knowledge, EMBASE, and IEEE Xplore. The search strategy covered three main areas, namely wearable technology, rehabilitation, and setting.

**Study selection:**

Potentially relevant studies were categorized as systems either evaluating methods or outcome parameters.

**Methods:**

Methodological qualities of studies were assessed by two customized checklists, depending on their categorization and rated independently by three blinded reviewers.

**Results:**

Twelve studies involving ABT met the eligibility criteria, of which three studies were identified as having implemented ABT for rehabilitation purposes in non-clinical settings. From the twelve studies, seven studies achieved high methodological quality scores. These studies were not only capable of assessing the type, quantity, and quality measures of functional activities, but could also distinguish healthy from non-healthy subjects and/or address disease severity levels.

**Conclusion:**

While many studies support ABT’s potential for telerehabilitation, few actually utilized it to assess mobility-related functional activities outside laboratory settings. To generate more appropriate outcome measures, there is a clear need to translate research findings and novel methods into practice.

## Introduction

A rapid increase in neurological disorders [1] has spurred contemporary healthcare services to reorganize themselves in response. Most neurological disorders cause functional disabilities due to motor impairments and physical deconditioning, giving rise to postural instability, gait disturbances, increased fall risks, mobility loss, increased fatigability, and reduced independence [2-4]. To improve individual health and well-being, rehabilitation programs aim to reduce or restore motor impairments and promote functional ability for those with neurological disorders. As many neurological disorders result in considerable morbidity [5], research on the effectiveness of neurorehabilitation for subjects with neurological disorders is of utmost importance, not only for those with disabilities but also for caregivers, treatment providers, policy makers, and society as a whole [6].

Presently, healthcare services face several widespread barriers, for instance, inadequate policies, standards, funding, information, communication, accessibility, and resources [7,8]. Studies on the integration of rehabilitation services, new technologies, pharmaceuticals, community exercise programs, and chronic disease management for people with neurological impairments have the potential to discover ways of improving outcomes while reducing costs.

The World Health Organization framework of International Classification of Functioning, Disability, and Health (ICF) employs a biopsychosocial model to describe human functioning through the capture of body function and through the individual’s activity and participation within his or her social and physical environment. It is important to note that in the activity and participation construct, there is a distinction made between a person's ability to perform a skill in the clinic and his or her ability to perform that same skill in a natural environment. The measurement of function, activities, and participation in natural environments is now possible through microelectromechanical systems (MEMS) and should be taken into consideration.

Nowadays, MEMS technology—particularly inertial sensors that contain accelerometers, gyroscopes, and occasionally magnetometers—can assess the type, intensity, duration, frequency, and quality of various mobility-related functional activities [9]. These sensing systems can be used to provide telerehabilitation, that is, the option to deliver rehabilitative services at remote sites, thereby introducing new possibilities for continuous, unsupervised, objective monitoring of mobility and functional activities in clinical [10] and non-clinical settings [11].

Numerous systematic and non-systematic reviews on telerehabilitation [11-15], physical activity monitoring [16-22], and human motion analysis [10,18,22-24] exist today. Some provide in-depth overviews of different motion-sensing applications for gait and balance evaluation, fall risk assessment, and mobility monitoring in various populations (e.g. stroke, elderly) in clinical [21,22] and non-clinical settings [19,20], ultimately verifying the feasibility of adopting wearable motion-sensing technology. Yet no reviews have evaluated the employment of valid motion-sensing technology capable of assessing functional activities in home and community settings within neurological populations. A clear understanding of translational research on existing motion-sensing technology validated for the community would provide a better grasp of what is and needs to be done to close the gap between basic science and practice [25].

The aim of this review is therefore to explore wearable accelerometry-based technology (ABT) capable of assessing the type, quantity, and quality of mobility-related functional activities in neurological populations in home and community settings. It will do so by addressing the following questions. Which sorts of accelerometry-based methods can accurately assess functional activities? Which types of outcome measures are suitable for obtaining quality measures of functional activities? Have these methods been implemented in home and community settings for rehabilitative purposes?

## Methods

### Study characteristics

Neurological disorders are categorized as major chronic diseases [26]. Because of their immense variety, this review only focuses on the most frequently occurring chronic conditions that induce motor fluctuations and movement disorders: Parkinson’s disease (PD), Multiple Sclerosis (MS), stroke, Cerebral Palsy (CP), and Huntington’s disease (HD).

MEMS technology offers various wearable motion-sensing applications, ranging from accelerometers, gyroscopes, and force-sensing resistors to inertial measurement units (IMUs). Because MEMS-based accelerometers form the basis for many motion-sensing applications, this review only considers wearable technology that contains accelerometers—at times, in conjunction with other MEMS applications. Accelerometers, in short, form a necessary but by no means sufficient criteria condition.

The ICF does not provide a unified definition of functional activities. Neither does general literature. The ICF does define mobility as changing (d410-d429) and maintaining body positions (d450-d469) [27]. Functional activities in this review denote the basic functional abilities considered key for independent living, such as walking, sitting, standing, and activity transitions.

### Literature search

A literature search was conducted on the following electronic bibliographic databases from January 2012 to January 2013: Cochrane (1940-2013), EMBASE (1974-2013), PubMed (1950-2013), Web of Knowledge (1980-2013), and IEEE Xplore (1946-2013). References from retrieved articles were checked using the Web of Science database. Generic search and MeSH terms for each database were used to identify relevant studies (Additional file 1: Appendix A). The search strategy was anchored on the following three categories: telemetry and rehabilitation, wireless technology, and human locomotion. Language restrictions were set. Only English studies were included.

### Study selection

**Figure 1:**
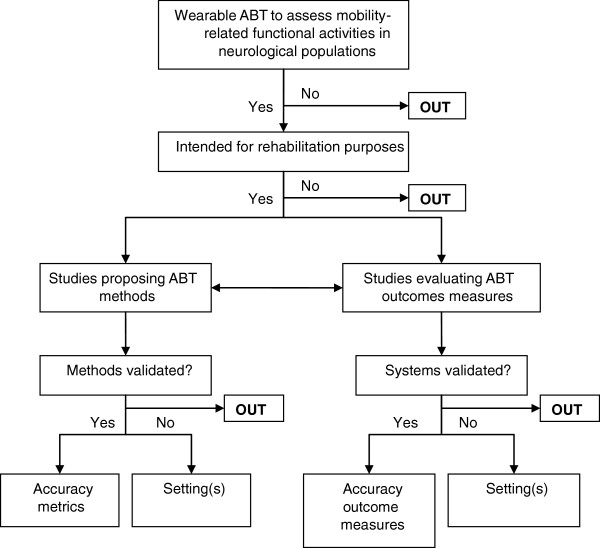
Procedure for the study selection and organization.

Figure 1 illustrates the selection procedure used to screen studies. For each database search, titles and abstracts were screened by three independent reviewers (DS, JC, and PE). Literature eligibility was initially determined by whether or not the title and abstract involved ABT for assessing mobility-related functional activities in neurological populations. The following exclusion criteria were used to further identify potentially relevant studies: a) telephone counseling interventions; b) network interventions; c) miscellaneous outcome measures, such as energy expenditure and activity behavior; d) miscellaneous technology used for assessing functional activities, such as robotics, pedometers, force-sensing resistors, virtual reality, and cueing devices; e) reviews; f) book reports; and g) off-topic articles. Studies that provided insufficient information to allow adequate interpretation of outcome measures and results were also excluded.

Full-text articles were then retrieved and evaluated by four independent reviewers (DS, HD, JC, and PE). Studies were included if they: a) concerned neurological conditions, b) employed wearable ABT, c) evaluated mobility characteristics of the lower extremity through functional activities, and d) were intended for rehabilitation purposes in home and community settings. Motor symptoms due to neurological disorders affecting mobility (e.g. spasticity, tremor) were additionally included in this review only if they were integrated as an aspect of mobility through functional activity testing. In order to ensure that results across studies were comparable, this review distinguished studies using ABT to evaluate the aforementioned outcome measures (mobility characteristics of functional activities) from studies proposing ABT approaches. The validation of ABT-systems served as the final screening measure for inclusion.

### Assessment of methodological quality

Three authors (HD, JC, and PE) independently evaluated the selected studies using two customized versions of methodological criteria adapted from the PEDro scale [28] and CONSORT [29] and Trend statements [30]. One version was founded on ABT that evaluates outcome parameters of functional activities, while the other drew upon ABT-methods.

Both customized versions were piloted to assess the reliability of the quality assessment process. All authors were blinded to paper authors, affiliations, publication dates, journals, funding sources, and references. Disagreements were resolved through consensus meetings.

The next step of the quality assessment process involved extracting information regarding the content, construction (e.g. measurement protocols), population (e.g. population size, reports of baseline characteristics), and measurement properties of each system. Extracted measurement properties were: content and criterion validity. The quality of measurement properties was determined by internal validity components (e.g. sample size) as well as external validity components (e.g. generalization). Study results needed to be founded on statistical methods, including reports of accuracy metrics.

Accelerometry-based outcome measures were deemed valid if the methods were cross-validated with a “gold standard” criterion, such as an optical motion camera system. Going further, studies that introduced ABT-methods based on activity classifiers or other approaches were also considered valid if their output successfully compared with that of suitable population-specific questionnaires (e.g. UPDRS) and/or cross-validated with appropriate statistical analysis (e.g. *K*-fold cross-validation, bootstrap method, leave-one-out method).

## Results

### Search

**Figure 2:**
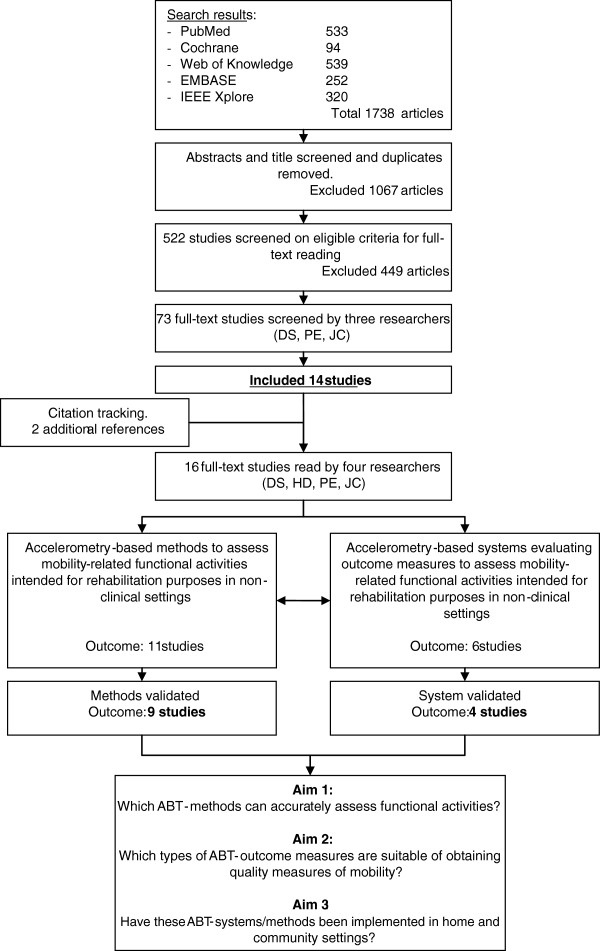
Flowchart of the results from the literature search.

**Figure 3:**
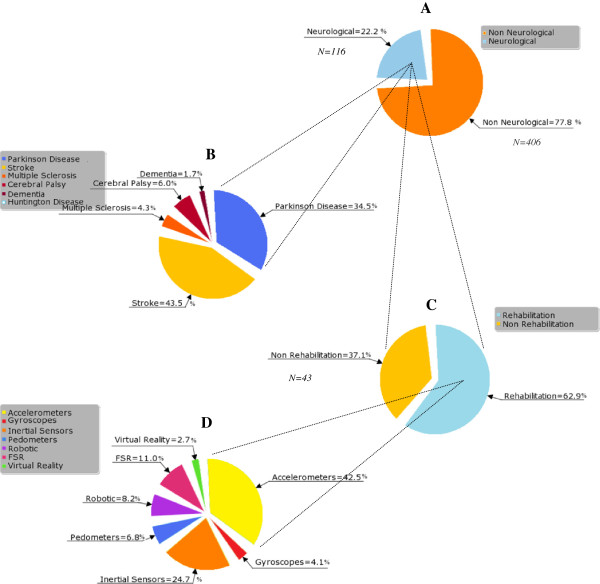
**Piechart of the screening results from the literature search.** Studies are divided in: **(A)** motion-sensing technology to assess functional activities in neurological or non neurological conditions; **(B)** type of neurological conditions; **(C)** technology intended for rehabilitation purposes; and **(D)** type of technology.

The initial literature search resulted in the retrieval of 1738 studies (Figure 2). After screening for relevant titles and abstracts, then winnowing out duplicates and off-topic studies, 522 studies remained. Most studies were excluded on the basis that they did not involve neurological conditions (Figure 3). In other cases, when authors published several studies on the same research initiative, only their most recent studies that satisfied the inclusion criteria were kept. After such selective factors were applied, 14 studies remained. A reference search on the Web of Science retrieved two more relevant studies. The now 16 studies [31-46] were categorized according to whether they proposed ABT-methods (*N* = 11), evaluated ABT-outcome measures (*N* = 6) able to assess mobility characteristics of functional activities, or performed a combination of both. A total of 12 studies passed the validation screening and were finalized for this review: nine method studies [31-34,38,39,42,43,45], four outcome studies [33,37,40,44], in which one study [33] straddles both categories. A total overview of included studies can be found in Additional file 2: Appendix B and Additional file 3: Appendix C.

### Study quality

#### External and internal validity

The methodological quality scores for those studies evaluating outcome measures for mobility-related functional activities were consistently high. Scores ranged from 10 to 12 (max 12), whereas quality scores for studies proposing ABT-methods were generally lower, ranging from 4 to 9 (max 13; Tables 1 and 2).

**Table 1:** Checklist for quality review of studies evaluating ABT-outcome measures

**Criteria**	**Dobkin et al**[33]	**Prajapati et al**[40]	**Zampieri et al**[44]	**Mizuike et al**[37]
**Customized scale items**				
**External validity**				
1. Eligibility criteria specified	1	1	0	1
**Internal validity**				
2. Baseline characteristics described	1	1	1	1
3. Measurement protocol clearly described	1	1	1	1
4. Measurement procedure is clearly described for each group to allow replication	1	1	1	1
5. Completely defined pre-specified outcome measures	1	1	1	1
6. Outcome measures are reliable and valid	1	1	1	1
7. Statistical methods used to compare groups outcomes	1	1	1	1
8. Between-group statistical comparisons are reported for at least one outcome	1	1	1	1
9. The study provides measures of variability for at least one outcome	1	1	1	1
10. Methods for additional analyses, such as subgroup analyses and adjusted analyses	0	0	1	1
11. Reported trial limitations	0	1	1	1
12. Interpretation of the results	1	1	1	1
**Total score**	**10**	**11**	**11**	**12**

**Table 2:** Checklist for quality review of studies proposing ABT-methods

**Criteria**	**Salarian et al**[42]	**Motoi et al**[39]	**Zwartjes et al**[45]	**Lau et al**[34]	**Barth et al**[31]	**Yang et al**[43]	**Cancela et al**[32]	**Moore et al**[38]	**Dobkin et al**[33]
**Customized scale items**									
**Internal validity method**									
1. Baseline characteristics described	0	1	0	1	1	0	0	1	1
2. System and devices are clearly described	1	1	0	1	1	1	1	1	1
3. Measurement protocol is clearly described for each group to allow replication	1	0	1	1	1	1	0	1	1
4. Methods of analysis clearly described	1	1	1	1	1	1	1	1	1
5. Classifier(s) are evaluated	1	n/a	0	1	1	n/a	1	n/a	0
6. Statistical methods used to test reproducibility	1	0	1	1	1	0	0	0	1
7. Reported accuracy metrics	1	0	1	1	1	0	0	0	0
8. Reported confidence intervals for classifier performance	0	n/a	0	0	0	n/a	0	n/a	0
9. Study limitations described	0	0	1	0	0	1	0	0	0
10. Interpretation of the results	0	0	1	0	1	1	0	1	1
**Construct validity method**									
11. Content validity	1	0	1^48^	0	0	0	1	0	0
12. Criterion-related validity is obtained	1	1	0	1	0	1	0	1	1
13. Cross-validation (i.e. test and training set)	1	0	1	1	1	0	0	0	0
**Total score**	**9**	**4**	**8**	**9**	**8**	**6**	**5**	**6**	**7**

Studies evaluating outcome measures of mobility-related functional activities largely involved stroke (*n* = 3) [33,37,40] and PD (*n* = 1) [44]. Stroke studies showed great diversity in study design, population demographics (e.g. population size, disease onset), and methodology. They consisted of pilot studies (*n* = 2) [33,44], one cross-sectional study [37], and one experimental study [40]. Across the stroke studies, age range was fairly comparable. Population demographics, conversely, were not comparable between studies, because of insufficient information. Most studies used a control group (*n* = 3) [30,34,41], of which only one adopted an age-matched control group [44]. System measurement properties were determined through internal validity components, which were stopwatch measures [33], optical motion-sensing systems [34,41], or footswitches [40]. Outcome measures were reasonably similar, mainly focusing on correlation and accuracy levels.

Studies evaluating ABT-methods were mainly PD studies (*n* = 6) [31,32,38,42,43,45] and, to a lesser extent, stroke (*n* = 3) [33,34,39]. None involved other neurological conditions. PD and stroke method studies were all based on experimental study designs with small population groups (*n* ≤ 10), minus two PD studies [28,29], which recruited testing groups of 20 subjects or more. Between stroke studies, the population age ranged widely (45-68 years). Some studies were conducted without a control group altogether [34,39]. None featured an age-matched control group [33]. The report of baseline characteristics and eligibility criteria revealed much diversity in medication treatment, disease onset, and disease severity (e.g. UPDRS scores), whether or not patients were sensitive to deep brain stimulation and experienced motor fluctuations. The measurement properties used to determine validity were primarily based on external validity components cross-validated with video recordings [35,40] and statistical methods, such as *K*-fold cross-validation [31,34]. Three studies employed a combination of internal and external validation procedures [29,39,42].

All studies demonstrated notable variation in terms of methodology, study design, population demographics, and outcome measures, making it difficult to evaluate study results, which subsequently made a meta-analysis unfeasible. For ease of reading, the results will be chronologically described according to the stated aims of this review.

#### Accelerometry-based methods able to accurately assess functional activities

The term “accuracy” was applied in two ways. Firstly, it was used to describe if ABT-methods were able to distinguish healthy from non-healthy subjects and assess disease severity levels. Secondly, the term was used to signify the precision of reported metrics.

As previously mentioned, nine studies [31-34,38,39,42,43,45], consisting of six PD studies [31,32,38,42,43,45] and three stroke studies [33,34,39], proposed valid accelerometry-based methods able to identify mobility-related functional activities. These methods were founded on various machine-learning classifiers (e.g. decision trees, support vector machines [SVM]) [31-34,42,45], algorithms [38,43], and gait cycle parameters [39]. Table 3 provides a detailed overview of the different methodological approaches used to determine the types of functional activities and their capabilities.

**Table 3:** Overview of accelerometry-based methods

**Authors**	**Population**	**Method**	**Validity**	**Quality**	**Quantity**	**Activity**
Lau et al [34]	Stroke	SVM, MLP, RBF	Leave-one-subject-out method	-	-	Walking
Barth et al [31]	PD	Boosting with decision stump as weak learner, LDA, and SVM with linear and RBF kernel	Leave-one-subject-out method	x	x	Walking, foot circling, and heel-toe tapping
Cancela et al [32]	PD	*k*NN, Parzen, Parzen density, binary decision tree, Bpxnc train NN by back-propagation, and SVM	Cross-validation	x	-	Daily activities (i.e. walking, lying, sitting, drinking a glass of water, opening and closing a door)
Salarian et al [32]	PD	Logic Regression model with Mamdani fuzzy rule-based classifier	Cross-validation	-	x	sit-to-stand and stand-to-sit
Zwartjes et al [45]	PD	Decision tree	Leave-one-subject-out method	x	-	lying, sitting, standing, and walking
Yang et al [43]	PD	Autocorrelation method	Video recordings	-	x	Walking
Motoi et al [39]	Stroke	Sagittal angle changes		-	-	Walking and sit-to-stand
Moore et al [38]	PD	Mathematical step-length algorithm	Pen techniques and video recordings	x	x	Walking
Dobkin et al [33]	Stroke	Naive Bayes classifier in combination with Gaussian discretization followed by a maximum likelihood estimation	Stopwatch	-	x	Walking

*Abbreviations:LDA* Linear Discriminant Analysis, *SVM* Support Vector Machines, *RBF* radial basis function neural network, *K-NN* K-nearest neighbour, *NN* Neural Network, *MLP* multi-layer perception; Quality, methods assessing severity levels, Quantity, methods able to distinguish healthy from non-healthy subjects.

##### Parkinson disease

*Classifiers.* Four out of the six PD studies [28,29,39,42] employed machine-learning classifiers. Three of these four used their classifier to identify ambulatory activities [31,32,45] and other functional activities (e.g. sitting, standing [32,45]). The remaining study used its activity classifier to detect sit-to-stand (STS) transitions from non-transitions [42]. From these four studies, Barth et al. [31] and Cancela et al. [32] evaluated various activity classifiers. Barth et al. [31] evaluated the following activity classifiers to detect gait patterns able to distinguish healthy controls from PD patients and mild gait impairments from severe ones: Boosting with decision stump (i.e. one-level decision tree), Linear Discriminant Analysis (LDA), and SVM with linear and Radial Basis Function (RBF) kernel. The accuracy of their sensor system was based on three activities from the UPDRS (Part III), namely 10 m walking, heel-toe tapping, and foot circling. The LDA classifier achieved the best overall accuracy, classifying patients and controls with a sensitivity of 88% and specificity of 86%. When optimized for the most accurate features, it reached a 100% sensitivity and specificity. The most optimal features were derived from step features (step duration), signal sequence (entropy [47], variance), and frequency analysis (energy ratio and 0.5-3 Hz energy band). Cancela et al. [32], on the other hand, used six different activity classifiers (i.e. *k*-nearest neighbour [*k*NN], Parzen, Parzen density, Binary decision tree, feed-forward neural network [Bpxnc], SVM) to automatically detect the severity of walking-derived bradykinesia according to UPDRS scores. The SVM classifier related the best to the UPDRS output scores by clinicians, with an accuracy range between 70 and 86% (i.e. sensitivity), using two statistical features (root mean square values and range) over a 5 s interval with 50% overlap.

Besides evaluating activity classifiers, Cancela et al. [32] also, like Zwartjes et al. [45], assessed symptom severity levels in PD as part of their detection process. Zwartjes et al. [45] employed a decision tree for a complete motor assessment by simultaneously analyzing various functional activities and symptom severity (i.e. tremor, bradykinesia, hypokinesia) at different levels of deep brain stimulation, with an overall accuracy of 99.3%. The motor assessment was mainly founded on UPDRS-items (Part III), including foot-tapping and several daily activities. Their PD monitor correlated well with the UPDRS and could, thus far, detect significant changes in rest and kinetic tremor, with an accuracy ranging from 78.7% to 94.1% depending on the activity performed.

The remaining study by Salarian et al. [42] used an activity classifier to categorize STS transitions. Able to separate transitions from non-transitions and differentiate between sit-to-stand and stand-to-sit with a sensitivity of 83.8% in PD and 94.4% in healthy controls, their method used a mamdani fuzzy rule-based classifier in tandem with two statistical classifiers based on a generalized logistic regression model. Acceleration and tilt measures of the trunk, previously described by Najafa [48,49], were used to detect transitions. Salarian’s method [42] has been integrated into the iTUG (instrumented Timed-Up and Go Test) [44,50], which also contains a 180° turn analyzing algorithm [51].

The two other PD studies [38,43] based their method on algorithms. Yang et al. [43] validated an autocorrelation function of the vertical acceleration for possible real-time analysis of disabling PD gaits for ambulatory rehabilitation, gait assessments, and motor fluctuations. Moore et al. [38], conversely, developed a validated stride length algorithm able to accurately estimate stride lengths of healthy and PD subjects in their natural environment. The ensuing stride length measures exhibited a linear relationship to the actual stride length (*r* = .98) and obtained an improved accuracy (mean error ± 0.05 m) relative to previous techniques utilized [52,53]. In a follow-up study, the capabilities of the stride length monitor were extended with the detection of freezing events in PD [54].

##### Stroke

*Classifiers.* Two out of three stroke studies [33,34] employed machine-learning classifiers for the detection of ambulatory activities, whereas the remaining stroke study assessed functional activities through angle measurements [39].

In presenting a possible tool for pathological gait analysis, pattern recognition, and activity monitoring, Lau et al. [34] explored the performance of various classifiers (i.e. SVM, artificial neural network [ANN], RBF, Bayesian belief network [BBN]) in different walking conditions (i.e. level ground, stair ascent, stair descent, upslope, downslope) for stroke subjects with dropped foot. The SVM proved superior to other classifiers, achieving an overall accuracy of 92.9% to 96.8% for both groups and individuals. In addition, it distinguished stair ascent and descent from other conditions with 100% accuracy and classified all five conditions with 84% accuracy.

Dobkin et al. [33] successfully implemented a naive Bayes method to estimate the walking speed of stroke patients in home and community settings. Feature selection was based on time domain data (e.g. dominant frequencies, amplitudes, waveforms of acceleration, signal derivatives) that was converted into vector form. The estimation of outdoor walking speeds highly correlated with stopwatch-measured speeds (*r* = .98; *p* = .001), including repeated measures (*p* = .01).

Unlike the two studies above, Motoi et al. [39] presented a gait and STS analyzing method based on angle and acceleration patterns to determine the level of long-term care. Noticeable angle changes and fluctuations of the trunk, thigh, and knee were detected between different severity and care levels.

### Outcome measures suitable for obtaining quality measures of functional activities

Research on the effectiveness of neurorehabilitation is of upmost importance and necessitates the determination of appropriate outcome measures beforehand. Four out of six studies [33,37,40,44] (three stroke studies [33,37,40], one PD study [44]) passed the eligibility criteria (§2.3) and were categorized as valid (Table 4).

**Table 4:** Overview of accelerometry-based outcome measures

**Authors**	**Population**	**Outcome parameters**	**Validity**	**Quality**	**Quantity**	**Activity**
Dobkin et al. [33]	Stroke	Walking speed, bouts of walking, gait symmetry	Leave-one-subject-out method	-	x	Walking
Zampieri et al. [44]	PD	Stride length, stride velocity, cadence, peak arm swing velocity on the MAS, and turning velocity	Leave-one-subject-out method	-	x	Sitting, standing, walking, turning
Mizuike et al. [37]	Stroke	Accelerometers derivatives, raw RMS, normalized RMS, autocorrelation function	Cross-validation	x	x	Walking
Prajapati et al. [40]	Stroke	Walking bouts, total walking time, gait speed, number of steps, gait symmetry, swing symmetry, cadence	Cross-validation	x	-	Walking

*Abbreviations:MAS* most affected side; Quality, studies assessing severity levels; Quantity, studies able to distinguish healthy from non-healthy subjects.

#### Stroke

Walking speed is generally considered to be a significant, sensitive, and reliable marker of deficit severity and walking ability [55]. With this understanding in mind, Dobkin et al. [33] and Prajapati et al. [40] both affirmed walking speed as a sensitive outcome measure able to evaluate the effect of rehabilitation on movement quality and stroke severity indices. Dobkin et al. [33] found walking speed could be related to stroke severity and recovery, where patients who walked faster than 0.8 m/s could reach higher speeds under different walking conditions than those who walked below 0.8 m/s. Prajapati et al. [40] more or less confirmed this observation by finding a correlation between walking speed, balance impairment as measured by the Berg Balance Scale [56] (*r* = .60; *p* < .013), and walking period (*r* = .51; *p* = .045).

Mizuike et al. [37] proposed a different outcome measure, an acceleration-derived one, normalizing root mean square (nRMS) values of acceleration to forge a new measure by which to evaluate gait characteristics and form an index of treatment outcomes for rehabilitation. The values of the nRMS may serve as an indicator for the dynamics of walking patterns, reflecting motor recovery and gait abilities. nRMS values were also able to discriminate between groups (*p* < .01) and several Brunnstrom Stages (III-V, IV-V, *p* < .05).

#### Parkinson’s disease

Previous studies on the performance of the iTUG by Zampieri et al. [57] and Salarian et al. [50] determined sensitive outcome measures that formed the basis for the home feasibility study conducted by Zampieri et al. [44]. Peak arm swing velocity on the most affected side, average turning velocity, cadence, and peak trunk rotation were significantly slower in PD than control subjects and may potentially be used to detect disease progression and patient response to symptomatic and disease-modifying treatments. It is to be noted that the iTUG’s STS components (e.g. duration, range of motion, angular velocity) were the least reliable, whereas walking and turning components (e.g. stride length and velocity, cadence, peak arm swing velocity, turning velocity) were the most reliable. When assessed at home, Zampieri et al. [44] demonstrated that from the aforementioned outcome measures, stride velocity (*p* = .02) and length (*p* = .002) affected PD subjects significantly, resulting in slower and shorter steps.

### Accelerometry-based technology implemented in non-clinical settings

From this review’s twelve featured studies, only three [33,38,44] actually took measurements in both clinical and non-clinical environments. Two out of these three studies, both incidentally dealing with stroke, proposed ambulatory activity pattern estimations, one for stride length [38] and the other for walking speed [33]. The remaining study by Zampieri et al. [44], a PD one, investigated the possibility of implementing the iTUG in home environments. It was the only study in this review to broach this subject.

Results for remaining neurological studies were only garnered through clinical assessments carried out at hospitals or laboratory environments. Although all studies were intended for telerehabilitation purposes, only Salarian et al.’s [42] and Motoi et al.’s [39] methodological studies helped pave the way for their other studies that examined the implementation of MEMS-based accelerometers in non-clinical settings.

## Discussion

### Application of ABT in neurological populations

Search results indicate that a vast amount of literature exists (*N* = 522), especially in the engineering field, on wearable motion-sensing applications that assess functional activities. Only 22.2% of studies mentioned, however, use ABT in neurological populations. Only 9.7% of these targeted studies, in turn, are intended for rehabilitative purposes in non-clinical settings. Of this last group, the majority of studies focus on the classification and quantification of ambulatory activities in Parkinson or stroke survivors, excluding subjects coping with other neurological diseases. The quantification of mobility-related functional activities—walking, in particular—generates much information on a patient’s physical capabilities, recovery, and activity behavior. But it is ill-equipped to address the qualitative measures of an individual’s exercise performance, inevitably omitting those details clinicians require to adapt therapies and medications to individual needs.

Although this review has identified a few studies that evaluate outcome measures able to address rehabilitation’s impact on movement quality, the primary challenge still remains: how can more appropriate outcome measures be established, those more useful in determining each severity stage’s movement quality? While the main focus of this review lies in the accurate classification of various functional activities, future research would ideally analyze these activities beyond the scope of step counts and duration.

### Accuracy of ABT-methods

The number of studies (*N* = 12) that covers the measurement properties around reliability, validity, and responsiveness of accelerometry-based systems in neurological populations remains relatively low. Those methodological studies included that cover measurement properties may, in some cases, be compromised in terms of sample size. For those studies proposing methods based on activity classifiers [31-34,42], sample sizes range from 5 to 27 subjects. Such numbers are usually too small to generalize accuracy levels. Two out of such activity classifier-based studies, moreover, lacked a control group [32,34]. It is therefore important to evaluate activity classifier performance according to larger, homogeneous population sets that include an equal number of healthy and non-healthy participants [58].

How effective or well-performing a classifier is not only depends on its overarching study design, but also on the selected features and ABT accuracy. In general, difficulties evaluating a particular classifier’s success or failure stems from the relative silence regarding the feature extraction process, which depends on the analysis of movement patterns [59]. How ABT plays into classifier performance remains less obscure because well-canvassed in comparison to feature extraction. While generally considered to be an easy-to-use and inexpensive type of technology, ABT is prone to offset fluctuations, sensor noise, and estimation errors, which lead to integration drift [60]. Integrating a Kalman filter with received signal strength indicator (RSSI) measurements can drastically reduce this drift, increasing overall accuracy [61,62]. Blumrosen et al. [62] have recently assessed the feasibility of employing RSSI in coordination with ABT for body tracking and feature extraction purposes, establishing various criteria and analytical methods to facilitate this end. Although this type of technology is a future option for telerehabilitation, it is beyond the scope of this review.

Most studies wielding ABT in neurological populations for remote rehabilitation employ various machine-learning classifiers covering different aspects of neurorehabilitation, ranging from activity classification and symptom severity level assessment to long-term activity monitoring. While the question of which classifier is ideal for remote monitoring naturally follows, it currently cannot be addressed due to scant research in the field of telerehabilitation. This review did, however, identify studies that cross-examine the performance of different activity classifiers and their feature selections [31,32,34]. The SVM, LDA, and decision tree seem to perform better than their counterparts. The SVM, it must be added, was presented in the top ten most influential machine-learning algorithms [58].

### Potential of ABT in non-clinical settings

In the process of gathering studies deploying ABT intended for rehabilitation purposes in non-clinical settings, this review identifies several promising studies [33,44,45]. Dobkin et al. [33] presents a pilot study that grounds its feature extraction on a naive Bayes method in a Medical Daily Activity Wireless Network [9,63]. Its machine-learning algorithm can not only identify, quantify, and qualify different activities, but also assess patient’s activity behavior during the day. Naive Bayes (or simply "Bayes") is easy to construct and can be readily applied to large data sets, as the method does not need any complicated iterative parameter estimation schemes.

In terms of assessing mobility deficits in patients with early to mid PD, Zampieri et al. [44] successfully tests the feasibility of assessing the iTUG in home environments. Several papers [50,51,57] have contributed to this approach, including Salarian’s feature extraction paper on STS transitions [42]. Their method can not only distinguish between PD and healthy subjects, but also between “on” and “off” conditions in PD subjects. In terms of feature selection, their feature sets correlate reasonably well with the UPDRS, indicating that severity levels can potentially be judged and monitored.

Exploring this potential, Zwartjes et al. [45] actually classify PD symptoms in various functional activities and their severity, correlating their ABT method with UPDRS scores (e.g. tremor *r* = .87, *p* < .01) in line with other studies [64,65]. Strikingly enough, their PD monitor detects changes between different conditions of brain stimulation, whereas the UPDRS did not. The UPDRS is the most widely used instrument for measuring PD symptom severity and has excellent test-retest reliability for motor scores (*ICC* 0.90), but achieves only moderate to good reliability for symptom-based scales (*ICC* 0.69 - 0.88) [66]. That an ABT-based PD monitor not only correlates well with the UPDRS, but also allows for more sensitive readings of PD symptoms renders it an attractive measurement tool to assist the UPDRS.

So far, MEMS-based accelerometers, embedded with machine-learning algorithms, are deemed able to accurately assess various mobility-related functional activities and disease symptom severity levels. Yet the effectiveness of ABT in home-based rehabilitation regimens still has to be further examined. Zampieri et al. [44] most relevantly addresses such questions, drawing out significant links (e.g. testing location influences stride length and velocity). Whether the iTUG can easily be administered in non-clinical settings without supervision, in particular, remains a pressing issue, as the device requires proper set-up before tests.

## Limitations

The original database search included MesH terms of specific neurological diseases, which prevented a significant number of engineering articles from being considered due to their indexing method. That the IEEE Xplore database only permitted limited search term bindings posed an additional hurdle to widening the review’s scope. A broader search strategy was implemented at this stage, one eliminating neurological terms while still covering only those studies surrounding the use of wearable ABT for rehabilitation purposes. More relevant studies were consequently extracted. Many articles, especially engineering ones, did not provide clear or complete titles, abstracts, or research contexts with which to discern their relevance at first glance. Full texts often fared no better, giving rise to interpretative problems on multiple levels (e.g. methodology, intervention). Such hermeneutic struggles rendered the search to find eligible articles more difficult.

Because of the broad search strategy, strict eligibility criteria were implemented, weeding out seemingly relevant studies [54,64,67,68] from being reviewed. The only validated studies that qualified for review related to PD and stroke, all analyzing movement patterns of different functional activities, with an emphasis on quantitative and qualitative outcomes. No validated studies around other neurological diseases were identified.

## Conclusion

This systematic review focuses and clarifies the degree to which accelerometry-based motion-sensing technologies have been successfully implemented in the field of telerehabilitation. Extending and revising the insights set forward by Bonato et al. [69] and Patel et al. [15], among other studies, this review surveys today’s employment of ABT in neurological populations and draws out its limitations within telerehabilitative contexts heretofore unaddressed. By thoroughly and meticulously sifting through 1738 articles and identifying the few that actually utilize ABT-methods capable of remotely assessing functional activities in neurological populations, it assists researchers in making informed, time-sensitive decisions regarding which current methods to use in target populations and why. In this case, only twelve studies were determined to reliably assess functional activities in neurological populations, of which only three implemented ABT in home environments. As small as this number appears, it is a hard-won indication of the need for more versatile research that adopts or improves current ABT-methods in various populations. Dobkin et al. [9] point out how extensive research has been undertaken within engineering—initiatives that, bolstered by current advances in MEMS technology, are slowly fulfilling demands in telerehabilitation and telemedicine. As this review emphasizes, however, clinical and real-world research significantly lags behind its engineering counterpart.

The main challenges facing the deployment of ABT rest in: a) the difficulty in homogenizing a range of distinct research methods and features to realize the same aims, b) the use of appropriate outcome measures for movement quality assessments, and c) the lack of awareness surrounding ABT’s clinical usefulness. In order to address such challenges, research should keep the following three initiatives in mind. Firstly, research should set analyzing standards in different target populations, thereby allowing researchers to better justify any potential deviations from existing methods. Secondly, research should aim to employ appropriate outcome measures in order to obtain qualitative movement features. Researchers can distinguish healthy from non-healthy subjects and classify functional activities and symptom severity levels with relatively high accuracy, but hardly explore the qualitative dimension of motor performance. The ability to assess patient movement in clinical and non-clinical settings would permit researchers, clinicians, and caretakers within the areas of prevention, diagnostics, disease progression, telerehabilitation, and telemedicine to improve individual health and well-being with greater nuance. Thirdly, follow-up studies should incorporate a multidisciplinary approach, with their research findings translated for a wider audience and ABT’s clinical practicality directly promoted as a result.

## Competing interests

The authors declare that they have no competing interests.

## Authors’ contributions

DS carried out the study design, study selection process, data extraction, data interpretation, and manuscript drafting. HD, PE, and JC participated in the process of study selection, data extraction, and manuscript revision. All authors read and approved the final manuscript.

## Supplementary Material

Additional file 1: Appendix A Search strategy. Click here for file

Additional file 2: Appendix B Overview of studies proposing ABT-methods to assess mobility-related functional activities in neurological populations. Click here for file

Additional file 3: Appendix C Study characteristics. Click here for file
