# Supplementary material for: Wearable accelerometry-based technology capable of assessing functional activities in neurological populations in community settings: a systematic review
Source: J Neuroeng Rehabil. 2014 Mar 13;11:36. doi: 10.1186/1743-0003-11-36 (PMC4007563; doi:10.1186/1743-0003-11-36)
Supplement: Additional file 1: Appendix A — Search strategy. [file 1743-0003-11-36-S1.docx]

# Appendices

| **Databases***   1. Cochrane Database for Systematic Reviews (CDSR), all of the Cochrane library content, via www.thecochranelibrary.com, (1940 - January 1, week 1, 2013) 2. EMBASE via OvidSP, (1974 - January 1, week 1, 2013) 3. Web of Knowledge ISI (WoK), via http://apps.webofknowledge.com, (1980 - January 1, week 1, 2013) 4. Web of Science via WoK, (January 1, week 1, 2013) 5. Biosis via WoK, (January 1, week 1, 2013) 6. PubMed via NCBI (National Center for Biotechnology Information), (1950 - January 1, week 1, 2013) 7. MEDLINE via WoK and PubMed (1950 – January 1, week 1, 2013) 8. IEEE Xplore, including Individual Online Journals, IEEE/IET Electronic Library (IEL), VDE VERLAG Conference Proceedings (1946 – January 1, week 1, 2013) |
| --- |
| *All database searches were originally search on January 1^st^ 2012, with update searches carried out in January 1^st^ 2013 |

## Appendix A

### PubMed search strategy

The following search strategy for PubMed and adapted it for the other databases.

1. exp physical examination/ or exp motion/ or exp locomotion/ or exp mobility limitation/
2. (motion) adj5 (sens$ or track$ or captur$ or detect$).tw.
3. mocap/
4. ((activity or inertial or mobility or position) adj5 (sens$ or monitor$)).tw.
5. accelerometer$/ or accelerometry/ or pedometer$/ or inertial sensor$/
6. human locomotion/ or human movement/
7. 1 or 2 or 3 or 4 or 5 or 6
8. exp telemetry/ or telemetries/ or exp rehabilitation/ or telerehabilitation/ or teletherapy/ or telehealth/ or telemedicine/
9. ((community-based or long-term or home or ambulatory) adj5 (rehab$ or recovery or monitor$)).tw.
10. 8 or 9
11. exp wireless technology/
12. ((wearable or remote or portable or mobile) adj5 (system$ or device$ or monitor$)).tw.
13. 11 or 12
14. 7 and 10 and 13

### Web of Knowledge (including BIOSIS, MEDLINE since 1950, and Web of Science) search strategy

1. motion or locomotion or mobility NEAR/1 limitation.tw.
2. (motion) NEAR/1 (sens* or track* or captur* or detect*).tw.
3. mocap/
4. ((activity or inertial or mobility or position) NEAR/1 (sens* or monitor*)).tw.
5. accelerometer* or accelerometry or pedometer* or inertial NEAR/1 sensor*
6. human locomotion/ or human movement/
7. 1 or 2 or 3 or 4 or 5 or 6
8. telemetr* or rehabilitation or telerehabilitation or teletherapy or telehealth or telemedicine or mobile NEAR/1 health.tw.
9. ((community-based or long-term or home or ambulatory) NEAR/1 (rehab* or recovery or monitor*)).tw.
10. 8 or 9
11. wireless NEAR/1 technology.tw.
12. ((wearable or remote or portable or mobile) NEAR/1 (system* or device* or monitor*)).tw.
13. 11 or 12
14. 7 and 10 and 13

### Cochrane search strategy

1. exp motion/ or exp locomotion/ or exp mobility limitation/
2. (motion) NEXT (sens* or track* or captur* or detect*).tw.
3. mocap/
4. ((activity or inertial or mobility or position) NEXT (sens* or monitor*)).tw.
5. accelerometer*/ or accelerometry/ or pedometer*/ or inertial NEXT sensor*/
6. human locomotion/ or human movement/
7. 1 or 2 or 3 or 4 or 5 or 6
8. exp telemetry/ or telemetries/ or exp rehabilitation/ or telerehabilitation/ or teletherapy/ or telehealth/ or telemedicine/
9. ((community-based or long-term or home or ambulatory) NEAR (rehab* or recovery or monitor*)).tw.
10. 8 or 9
11. exp telecommunications/
12. ((wearable or remote or portable or mobile) NEAR (system* or device* or monitor*)).tw.
13. 11 or 12
14. 7 and 10 and 13

### EMBASE search strategy (OvidSP)

1. exp physical examination/ or exp motion/ or exp locomotion/ or exp mobility limitation/ or exp walking difficulty/
2. (motion) adj5 (sens$ or track$ or captur$ or detect$).ti,sh,hw,ab,kw,tw.
3. Mocap. ti,sh,hw,ab,kw,tw.
4. ((activity or inertial or mobility or position) adj5 (sens$ or monitor$)).ti,sh,hw,ab,kw,tw.
5. accelerometer$/ or accelerometry/ or pedometer$/ or inertial sensor$/
6. human locomotion/ or human movement/
7. 1 or 2 or 3 or 4 or 5 or 6
8. exp telehealth/ or exp telemetry/ or exp remote sensing/ or exp telemonitoring/ or rehabiliation/ or telerehabilitation.mp. or telemetries.mp. or exp teletherapy/ or exp telemedicine/ or exp mobile health/
9. ((community-based or long-term or home or ambulatory) adj5 (rehab$ or recovery or monitor$)).ti,sh,hw,ab,kw,tw.
10. 8 or 9
11. exp wireless technology/
12. ((wearable or remote or portable or mobile) adj5 (system$ or device$ or monitor$)).ti,sh,hw,ab,kw,tw.
13. 11 or 12
14. 7 and 10 and 13

### IEEE Xplore search strategy

1. exp motion/ or exp locomotion/ or exp mobility limitation/
2. (motion) NEAR (sensing or trackting or capturing or detecting).tw.
3. mocap/
4. ((activity or inertial or mobility or position) NEAR (sensors or monitors)).tw.
5. accelerometers/ or accelerometry/ or pedometers/ or inertial sensors/
6. human locomotion/ or human movement/
7. 1 or 2 or 3 or 4 or 5 or 6
8. exp telemetry/ or telemetries/ or exp rehabilitation/ or telerehabilitation/ or teletherapy/ or telehealth/ or telemedicine/
9. ((community-based or long-term or home or ambulatory) NEAR (rehabilitation or recovery or monitoring)).tw.
10. 8 or 9
11. exp wireless technology/
12. ((wearable or remote or portable or mobile) NEAR (systems or devices or monitors)).tw.
13. 11 or 12
14. 7 and 10 and 13
