# Supplementary material for: Wearable accelerometry-based technology capable of assessing functional activities in neurological populations in community settings: a systematic review
Source: J Neuroeng Rehabil. 2014 Mar 13;11:36. doi: 10.1186/1743-0003-11-36 (PMC4007563; doi:10.1186/1743-0003-11-36)
Supplement: Additional file 2: Appendix B — Overview of studies proposing ABT-methods to assess mobility-related functional activities in neurological populations. [file 1743-0003-11-36-S2.docx]

## Appendix B

| Studies proposing ABT-methods to assess mobility-related functional activities in neurological populations | | | | | | |
| --- | --- | --- | --- | --- | --- | --- |
| **Reference (Year)** | **Population/study design** | **Sensor system** | **Methodology** | **Validation procedure** | **Results** | **Correlation with clinical measure** |
| Barth et al^31^ (2010) | - Experimental study - Parkinson’s disease (*N*=27); Group 1 – mild PD, UPDRS Scores <15 (*N*=14); Group 2 – intermediate PD, UPDRS >20 (*N*=13) - Healthy controls (*N*=16) | Two inertial sensors (Shimmer sensor unit, Ireland) (attached on each lateral heel of a shoe)  Sample frequency: 100Hz | Distinguish mild and severe gait impairment   - using frequency, step, and sequence features from single steps and complete gait cycle   Three different classifiers tested:   - Boosting with Decision Stump as a weak learner - LDA - SVM with linear and RBF Kernel | The classification accuracy was assessed with a leave-one-subject-out method | The best overall accuracy was reached in each case using the LDA classifier.  Classification:  Control vs. Group 1: sensitivity of 88% and specificity of 86% (10- meter walk test).  Distinction of both Control vs. Group 2 and Group 1 vs. Group 2 reached a sensitivity and specificity of 100% | __ |
| Cancela et al^32^ (2010) | - Experimental study - Parkinson’s disease (*N*=20); stable dopaminerg treatment, experiencing motor fluctuations - Healthy controls (*N*=16) | Six ALA-6g tri-axial accelerometers (ANCO, Greece) (attached on the limbs, trunk, and belt)  Sample frequency:  *unknown* | Determine severity of bradykinesia   - using the resultant vector (Euclidean vector method) - Statistical features: RMS, entropy, range of values and cross correlation in 12 different combinations   Six classifiers used to classify the epochs:   - *k*NN classifier - Parzen classifier - Parzen density based classifier - Binary decision tree - Bpxnc Train NN classifier - SVM   Method output according to the UPDRS, score between 0 and 4 | Clinician UPDRS scores  Classifier - Cross-correlation method (training and test set) | Symptom severity accuracies lay within the range of 70% - 86% | Related output scores to the UPDRS, but no correlation measures |

Abbreviations: RMS, Root Mean Square; UPDRS, Unified Parkinson Disease Rating Scale; LDA, Linear Discriminant Analysis; RBF, Radial Basis Function; K-NN, K-nearest neighbour; NN, neural network; SVM, Support Vector Machine; f/s, frames per seconds

| Studies proposing ABT-methods to assess mobility-related functional activities in neurological populations | | | | | | |
| --- | --- | --- | --- | --- | --- | --- |
| **Reference (Year)** | **Population/study design** | **Sensor system** | **Methodology** | **Validation procedure** | **Results** | **Correlation with clinical measure** |
| Dobkin et al^33^ (2011) | - Pilot study - Stroke (*N*=12) - Healthy controls (*N*=6) | Two tri-axial accelerometers (*unknown*) (attached above the ankle)  Sample frequency: 320Hz | Detection and activity classification   - NB classifier   Features: vector derived from frequencies, amplitudes, and waveforms of accelerations and decelerations, and time averages and derivatives   - Gaussian discretization of features into model-free clusters, followed by maximum likelihood estimation for real classification | Stopwatch measures, observer step counts, and activity logs | Concurrent validity comparison between stopwatch timed and algorithm-derived outdoor walking speeds, as well as their relationship to the indoor walking speeds  Walking speed  Stroke group:   - Pearson correlation outdoor walking speed r = .98 (*p*= .001) - Retest-reliability for repeated walks was high (*p* =.01)   Healthy group:   - Pearson correlation r = .98 (*p*= .001)   Activity detection   - All periods of mobility and lower extremity exercise were correctly categorized in healthy and stroke subjects | __ |
| Moore et al^38^ (2007) | - Experimental study - Parkinson’s disease (*N*=7) - Healthy controls (*N*=10) | One inertial sensor (MT9, Xsens, The Netherlands) (attached on the shank)  Sample frequency: *unknown* | Estimation of stride length by proposing a novel step-length algorithm based on the vertical acceleration and pitch angular velocity  - Least-square error fit | Pen technique  Comparison to video recordings | Accuracy stride monitor estimation, mean error 0.05m | __ |
| Motoi et al^39^ (2005) | - Experimental study - Stroke (*N*=14) - Healthy controls (*N*=11) | Two dual-axial accelerometers (ACA302, Star Micronics) and one inertial sensor (dual-axis acc. + single axis gyro [ENC-03J, Murata]) (attached on the sternum, thigh and calf)  Sample frequency:  25Hz | Detection of dynamic and static posture changes  Motion characteristics:   - Angle changes in sagittal plane - Walking speed | Comparison to video recordings 30f/s | Comparison wearable system accuracy against video recordings   - Walking speed; *r*=.992 - Angle change; *r*=.997   Comparison rehabilitation program before and after (no data presented)   - Before: more fluctuations maximum values angle changes - After: higher repeatability in detecting maximum values | __ |

Abbreviations: NB, Naive Bayes

| Studies proposing ABT-methods to assess mobility-related functional activities in neurological populations | | | | | | |
| --- | --- | --- | --- | --- | --- | --- |
| **Reference (Year)** | **Population/study design** | **Sensor system** | **Methodology** | **Validation procedure** | **Results** | **Correlation with clinical measure** |
| Lau et al^34^ (2009) | - Experimental study, observational classifier - Stroke (*N*=7) - No Control group | Two inertial sensors (one attached to the shank and one to the foot)  Sample frequency:  240 Hz | Test the performance of three different SVM classifiers (SVM, MLP, and RBF) – under 5 different walking conditions  (i.e. stair ascent, stair descent, level ground walking, upslope and downslope treadmill walking)   - 4 Classification tasks: - 5-class (all conditions separately) - 3-class (stair ascent, stair descent, and combined [level slope walking])   Feature extraction, dataset variables:   - Sh(AV_ps_) – pre-swing phase - Sh(Acc_ps_) – pre-swing phase - Ft(Acc_ps_) – pre-swing phase - Sh(Acc_is_) – initial swing phase - Ft (Acc_is_) – initial swing phase   Dataset 1: Shank variables  Dataset 2: Foot variables  Dataset 3: Shank and foot variables | Dataset: 50% training set and 50% testing set | The SVM technique always performed better than MLP and RBF  The overall accuracy increased from 92.9% to 96.8% in 3-class classification for a group and for an individual | __ |
| Salarian et al^42^ (2007) | - Experimental study - Parkinson’s disease (*N*=10) - Healthy controls (*N*=10) | Three inertial sensors (one attached to the trunk, two to the shanks)  Sampling frequency: 200Hz | Detection and classification of transition  Parameters used:   - TD (s) - Min (θ_g-lp_) - Range(θ_g-lp_) - Range(a_trunk-lp_), Max(a_trunk-lp_), Min(a_trunk-lp_), t{Min(a_trunk-lp_)}, t{Max(a_trunk-lp_)}   Separate transitions from non-transitions   - two statistical classifiers based on logistic regression model   Classification of the activities   - implemented a fuzzy classification method (6 fuzzy variables used) | Video comparison, event detection  Face to face validity with the UPDRS  Trained method (transition/non-transition): 70% data set (randomly) and 30% used for evaluation of outcomes | Detection of posture transitions, compared to video recording as a reference   - Sensitivity 94.4% (Controls) and 83.8 (PD) - Specificity 96.9% (Controls) and 87.0% (PD)   Classification of basic activities (i.e. walking, standing, sitting, and lying   - Sensitivity controls - walking (99.1%), standing (96.1%), sitting (99.5%), and lying (100%) - Sensitivity PD- walking (98.5%), standing (83.6%), sitting (86.3%), and lying (91.8%) | Significant correlation (*p*<.05) with the UPDRS:   - TD, *r*= .64 - Max(a_trunk-lp_), *r*=-.55 - Min(a_trunk-lp_), *r*=.69 - Range(a_trunk-lp_), *r*=-.65 |

Abbreviations: UPDRS, Unified Parkinson Disease Rating Scale; TD, duration or transition, defined as time interval between two peaks; Min (θ_g-lp_), amplitude negative peak trunk flexion/extension; Range(θ_g-lp_), range of antero-posterior tilt of the trunk; Range(a_trunk-lp_), Max(a_trunk-lp_), Min(a_trunk-lp_), t{Min(a_trunk-lp_)}, t{Max(a_trunk-lp_)}, the norm of the acceleration vector of the trunk sensor; SVM,Support Vector Machine; MLP, Neural network using multi-layer perceptron; RBF, radial basis function network; Sh(AV_ps_), shank angular velocity, Sh(Acc_ps_), amplitude values of the anterior-posterior acceleration

| Studies proposing ABT-methods to assess mobility-related functional activities in neurological populations | | | | | | |
| --- | --- | --- | --- | --- | --- | --- |
| **Reference (Year)** | **Population/study design** | **Sensor system** | **Methodology** | **Validation procedure** | **Results** | **Correlation with clinical measure** |
| Yang et al[^43^](#_ENREF_41) (2011) | - Experimental study - Parkinson’s disease (*N*=5); H&Y stage II and III - Healthy controls (*N*=5) | One tri-axial accelerometer (Kionix,KXPA4-2050) (attached to the trunk)  Sample frequency: 50Hz | Autocorrelation procedure  Gait cycle features obtained:   - Step regularity - Stride regularity - Step symmetry - Cadence | Cadence validation through video recordings | Cadence validation:  Mean absolute percentage error 4.89%  Comparison multiple slide windows (Mean CV, Mean error)  Cadence: 1.21%, 0.67%  Step regularity: 8.53%, 2.44%  Stride regularity: 9.34%, 4.47%  Step symmetry: 7.78%, 2.04% | __ |
| Zwartjes et al[^4^](#_ENREF_43)^5^ (2010) | - Clinical trial with control group - Parkinson’s disease (*N*=6); sensitive to DBS treatment (<5min), no major motor fluctuations due to medication - Healthy controls (*N*=7) | Four inertial MT9 sensors (Xsens Technologies, The Netherlands)  (attached on the trunk, wrist, thigh and foot of the MAS)  Sample frequency: 50Hz | Classify motor activity   - AC to differentiate between lying, sitting, standing, standing up, and walking - Binary decision nodes: 7 nodes to discriminate activities   Feature extraction for particular nodes   1. IAA thigh (change in thigh inclination) 2. IAA trunk (change in trunk inclination) – differentiate lying – standing-up 3. IAA thigh - differentiate standing – sitting 4. IAA wrist – differentiate active arm movement – no arm movement   MSM - Classify motor symptom severity (i.e. rest/ kinetic tremor, bradykinesia, and hypokinesia)  Tremor: feature extraction   - Frequency analysis - Algorithm TS = $i\sqrt{d}$   Bradykinesia: feature extraction   - average value of acceleration (during periods of AAM) - step length, step velocity - duration standing-up   Hypokinesia   - % arm movement during the entire sitting and standing time - Arm swing and thigh swing correlation | Compared to video recordings (25 f/s) – test-retest reliability not assessed | AC - Activity detection   - PD – Overall accuracy of 98.9% - Controls - Overall accuracy of 99.3%   MSM - Quantify tremors   - PD - accuracy of rest tremor; sitting (84.5%) and standing (94.1%) in the arm; sitting (79.1%) and standing (90.1%) in the thigh - Kinetic tremor in the arm detection; accuracy sitting (78.7%) and standing (81.7%)   Severity in tremors (rest/kinetic), which observed within one individual, often different within the arm, thigh, and trunk  Proposed method/monitor can discriminate between different settings of the DPS stimulator; Arm, thigh, and trunk rest tremor (*p*<.05, *p*=.01, and *p*<.01) | Quantification tremor with UPDRS 20 scores   - Best correlation - rest tremor in arm, *r*=.84 (*p*<.01) - Kinetic tremor, *r*=.67 (*p*<.01) up to UPDRS item 21 |

Abbreviations: H&Y, Hoehn & Yahr; AC, Activity Classifier; DBS, Deep Brain Stimulation; IAA, Integrals of the Absolute value of the Accelerometer output; MSM, Motor Symptom Monitor; AAM, Active Arm Movement; TS, tremor severity; MAS, Most Affected Side; CV, coefficient of variance

| Studies evaluating ABT-outcome measures to assess mobility-related functional activities in neurological populations | | | | | | |
| --- | --- | --- | --- | --- | --- | --- |
| **Reference (Year)** | **Population/study design** | **Validation procedure** | **Clinical outcome measure(s)** | **Gait parameters** | **Results** | **Correlation with clinical measure** |
| Dobkin et al^33^ (2011) | - Pilot study - Stroke (*N*=12) - Healthy controls (*N*=6) | Stopwatch calculations | 50-ft clinic walks at slow, casual, and fast speeds  300-ft clinic-outdoor walks | Gait speed  Bouts of walking  Cadence  Swing time | Pearson correlation between stopwatch measured indoor walking speed and algorithm-calculated speed, *r*=.98, *p*=.001 and for repeated measures (*p*=.01) | __ |
| Mizuike et al^37^ (2009) | - Cross-sectional study - Stroke (*N*=63); Brunnstrom stage III(10), IV(22), V(15), VI(16) - Control group (*N*=21); elderly | Recorded observations | 10m walk test | Accelerometers derivatives (x, y, z axis):  Raw RMS  Normalized RMS  Autocorrelation function | Raw RMS and AC values significantly lower and normalized RMS values were significantly higher at all axes in stroke patients compared to controls.  These parameters were also significantly different between control groups and each group in the different Brunnstrom motor recovery stages. | Brunnstrom Stages |
| Prajapati et al^40^ (2011) | - Experimental study - Subacute Stroke (*N*=16); BBS 41.8 ± 9.9 - No control group | Footswitch system – comparing FO and FC events | Compared laboratory gait assessment with GAITRite system | Walking bouts  Total walking time  Gait speed  Number of steps  Gait symmetry  Swing symmetry  Cadence | Significant association between the number of walking bouts to the total walking time (*r*=.76; *p*< .006) and laboratory gait speed (*r*=.51; *p*<.45)  Laboratory gait speed and BBS (*r*=.60; *p*<.013)  Significant gait symmetry increase between day-long measurement compared with standard laboratory assessment (*p*=.006), 12 out of 16 were more asymmetrical during the day-long measurement | Lower laboratory gait speed and lower BBS score |
| Zampieri et al[^44^](#_ENREF_44) (2011) | - Pilot study - Parkinson’s disease (*N*=6); early-to-mid stages, UPDRS 28.6 ± 15 and H&Y 1.9 ± 0.7 - Healthy controls (*N*=8) | Reported in Simoes et al.[^49^](#_ENREF_49) – compared to a Vicon system | iTUG test – 7m walkway  (three trails conducted at home and laboratory setting) | Stride length  Stride velocity  Cadence  Peak arm swing velocity  Turning velocity | - Distances walked at home were slower and with shorter steps in the PD group than the laboratory, but similar between groups: PD = 5.9 ± 0.5m, Control = 5.9 ± 0.6m in laboratory - Significant group effect for stride velocity (*p*= 0.03), cadence (*p*=.001), peak arm swing velocity MAS (*p*=.002), and turning velocity (*p*=.02) - Significant interaction effect for stride velocity (*p*=.02), and stride length (*p*=.002) - Significant location effect for turning velocity (*p*=.002) in control group | __ |

Abbreviations: UPDRS, Unified Parkinson Disease Rating Scale; H&Y, Hoehn & Yahr Score; iTUG, instrumented Timed Up and Go Test; MAS, most affected side; 5WMT, 5-min Walking Test; BBS, Berg and Balance Scale ;FO, Foot off; FC, Foot Contact
