# Supplementary material for: Wearable accelerometry-based technology capable of assessing functional activities in neurological populations in community settings: a systematic review
Source: J Neuroeng Rehabil. 2014 Mar 13;11:36. doi: 10.1186/1743-0003-11-36 (PMC4007563; doi:10.1186/1743-0003-11-36)
Supplement: Additional file 3: Appendix C — Study characteristics. [file 1743-0003-11-36-S3.docx]

## Appendix C

| **Study Characteristics** | | | | | | | | |
| --- | --- | --- | --- | --- | --- | --- | --- | --- |
| **Reference (Year)** | **Population** | **n (E/C)** | **Mean age, years (range) or SD** | **Male/Female** | **Intervention** | **Setting** | **Intended for** | **Medication** |
| Barth et al^[61]^ (2011) | PD | 27/16 (healthy controls)  Group I: 14  Group II: 13 | Group I: 63.4 ± 9.3 / 64.9 ± 6.9  Group II: 66.6 ± 10.5 | 12/2 – 7/9  9/4 – 7/9 | - | Laboratory | Smart home monitoring | Levodopa (mg)  Group I: 408 ± 415  Group II: 563 ± 359 |
| Cancela et al^[63]^ (2010) | PD | 20/- | - , (18-85) | - | - | Supervised environment (clinic) | Telehealth, Telemedicine, Home monitoring | - |
| Dobkin et al^[53]^ (2011) | Stroke | 12/6 (healthy controls) | 58.9 ± 12.6 / 40.0 ± - | 6/6 – 3/3 | - | Clinic indoor to outdoor, Home setting | Rehabilitation, Community | - |
| Lau et al^[60]^ (2009) | Stroke | 7/- | 45.6 ± 5.4 | 5/2 | - | - | Rehabilitation, Home monitoring | - |
| Mizuike et al^[56]^ (2009) | Stroke | 63/21 (healthy controls elderly) | 69.4 ± 10.2 / 74.8 ± 6.9 | 46/17 – 4/17 | - | Hospital | Rehabilitation | - |
| Moore et al^[64]^ (2007) | PD | 7/10 | 72.0 ± 7.4 / 38.0 ± 7.7 | 3/4 – 5/5 | - | Clinic (indoor to outdoor), Home setting | Telerehabilitation and medication | - |
| Motoi et al^[58]^ (2005) | Stroke | 7/- | 68.6 ± 10.2 | 4/3 | - | Laboratory | Rehabilitation |  |
| Prajapati et al^[54]^ (2011) | Stroke | 16/- | 59.74 ± 15.3 | 12/4 | - | Rehabilitation hospital | Home monitoring | - |

Abbreviations: SD, Standard deviation

## Appendix C (continued)

| **Study Characteristics** | | | | | | | | |
| --- | --- | --- | --- | --- | --- | --- | --- | --- |
| **Reference (Year)** | **Population** | **n (E/C)** | **Mean age, years (range) or SD** | **Male/Female** | **Intervention** | **Setting** | **Intended for** | **Medication** |
| Salarian et al^[57]^ (2007) | PD | 10/10 (healthy controls) | 61.5 ± 7.8 / 63.6 ± 10.5 controls age- and gender matched | 5/5 – 5/5 | STN-DBS – 45min protocol; once STIM “On” and once STIM “Off” | Laboratory | Telemedicine, Home monitoring | - |
| Yang et al^[62]^ (2010) | PD | 5/5 (healthy controls) | 78.0 ± 9.8 / 26.0 ± 3.1 | 4/1 – 5/0 | - | Laboratory | Rehabilitation, Teleheath | - |
| Zampieri et al^[55]^ (2011) | PD | 6/8 (healthy controls) | 57.3 ± 8.6 / 63.7 ± 5.9 | 3/3 – 2/6 | - | Home/Laboratory | Home monitoring | - |
| Zwartjes et al^[59]^ (2010) | PD | 6/7 (healthy controls) | - ,(54-68) / - ,(53-61) | - | DBS (3 different levels) – various activities, incl. UPDRS tasks (2x)  Condition 1 ("on"): Stim. at the optimal setting  Condition 2 ("intermediate"): Stim. at stimulation amplitude of 80% of the optimal setting  Condition 3 ("off"): Stimulator off | Laboratory | Ambulatory environment | - |

Abbreviations: SD, Standard deviation; STN-DBS, Sub thalamic Nucleus - Deep Brain Stimulation; DBS, Deep Brain Stimulation
